# Synthesis and Properties of a Novel Four-Coordinate 8-Hdroxy-Quinolate-Based Complex

**DOI:** 10.3390/ijms262110528

**Published:** 2025-10-29

**Authors:** Jun Cheng, Zilong Zhang, Ruiyao Wang, Hongbo Wang

**Affiliations:** 1 National Center for International Cooperation and Disciplinary Innovation in Sustainable Chemical Engineering, Key Laboratory of Flexible Optoelectronic Materials and Technology (Ministry of Education), School of Optoelectronic Materials and Technology, Jianghan University, Wuhan 430056, China; juncheng@liverpool.ac.uk (J.C.); z1159741412@163.com (Z.Z.); 2 XJTLU Wisdom Lake Academy of Pharmacy, Xi’an Jiaotong-Liverpool University, 111 Ren’an Road, Suzhou 215123, China; 3 Department of Chemistry, University of Liverpool, Crown Street, Liverpool L69 7ZD, UK

**Keywords:** annihilation electrochemiluminescence, boron, four-coordination, 8-hydroxyquinoline

## Abstract

Organoboron compounds have attracted significant interest because of their special properties conferring photoluminescence, electroluminescence, and chemiluminescence. However, there are few reported examples of their use in electrochemiluminescence (ECL). This work represents a significant contribution by providing comprehensive evidence that elucidates the critical role of four-coordinate boron coordination in modulating and enhancing ECL properties. By introducing a coordinatively saturated boron center into a designed molecular framework, we achieved a 2.5-fold enhancement in ECL intensity during electrochemical processes. These results address the limited fundamental understanding of ECL behavior pertinent to tetracoordinate boron systems and provide valuable insights into molecular design strategies for developing stable and highly efficient ECL luminophores.

## 1. Introduction

Electrochemiluminescence (ECL), a light-emitting phenomenon driven by synergistic electrochemical and chemiluminescent processes, has revolutionized analytical chemistry through its unparalleled sensitivity, minimal background interference, and precise spatiotemporal control over signal generation [1,2,3]. These attributes have positioned ECL as a cornerstone technology for detecting trace analytes in biomedical diagnostic, environmental monitoring, and food safety applications [3,4,5,6,7]. Central to optimizing ECL performance is the development of luminophores that combine high photoluminescence quantum yields with stable redox-active intermediates capable of sustaining efficient electron transfer cycles. Diverse ECL luminophores have been explored, including inorganic luminophores [8,9], organic molecules [10,11,12], and nanomaterials [13,14]. Notably, organic emitters offer distinct advantages, including structural versatility and synthetic tunability [15].

Among the organic luminophores, tetracoordinate boron-nitrogen complexes derived from 8-hydroxyquinoline (8-HQ) and its derivatives have emerged as a paradigm shifting class of materials since the seminal work on tris(8-hydroxyquinolinato)-aluminium by Tang and VanSlyke, which laid the foundation for organic light-emitting diodes (OLEDs) [16]. The versatility of 8-HQ derivatives stems from their robust coordination chemistry, tunable electronic structures, and efficient luminescence properties that are further amplified when integrated with electron-deficient boron centers [17]. The tetracoordinate boron atom, with its vacant p-orbital, enhances electron delocalization within conjugated frameworks to facilitate charge transfer processes, which enables its optical and redox properties to be precisely modulated through structural engineering [18]. For example, Yamaguchi et al. demonstrated that tetracoordinate boron enhances luminescence efficiency by planarizing molecular backbones and reducing LUMO levels through B–N coordination [19].

While tetracoordinate boron complexes offer attractive features for luminescent material design, most studies beyond boron dipyrromethene derivatives have focused primarily on photoluminescence properties [20], with limited exploration of their electrochemical and ECL behaviors. This gap persists owing to challenges including complex precursor synthesis, difficult purification, and aggregation-caused quenching [21,22].

In this work, we synthesized an 8-hydroxyquinolate derivative (L1) that, when coordinated with triphenylborane, led to the isolation of a four-coordinate boron complex (P1). The photophysical characteristics, electrochemical attributes, and electrogenerated chemiluminescence of L1 and P1 were investigated. A comparative analysis of pre- and post-coordination ECL performance, supported by theoretical and experimental evidence, revealed significant modulation of these properties. These insights established the foundational principles for molecular engineering of boron-containing four-coordination complexes.

## 2. Results

### 2.1. Design and Synthesis of the Four-Coordinate Emitter

Planar, conformationally locked π-systems featuring ring-fused architectures have garnered significant interest due to their excellent photophysical characteristics, including intense emissions, thermal resilience, and high charge transport capabilities [23,24]. Strategically incorporating main-group heteroatoms (e.g., N, B, and P) into these conjugated frameworks constitutes a powerful means of tailoring optoelectronic signatures [25,26]. Numerous quinoline derivatives have been engineered with rigid fluorescent cores by forming Lewis acid-base adducts with triphenylborane, with broad utility in OLED technologies [21,27,28,29]. However, the impact of this structural transformation on ECL behavior remains largely unclear. To address this knowledge gap, we synthesized a tailored quinoline derivative and reacted it with BPh_3_ to generate a tetracoordinate boron complex. This molecular pair was designed to enable a systematic investigation of coordination-induced electronic restructuring and radiative property changes.

**Scheme 1:**
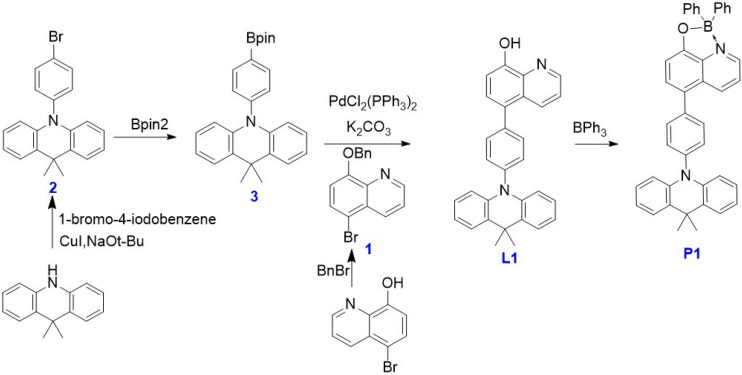
The synthetic route of products L1 and P1.

As shown in Scheme 1, the synthetic sequence commenced with benzylation of 5-bromo-8-hydroxyquinoline using benzyl bromide, affording the protected intermediate 1 (5-bromo-8-benzyloxyquinoline), following procedures previously described in the literature [28,30]. Concurrently, the donor moiety 3 (9,9-dimethyl-10-[4-(4,4,5,5-tetramethyl-1,3,2-dioxa-borolan-2-yl)phenyl]-9,10-dihydroacridine) was synthesized from 9,9-dimethyl-9,10-dihydroacridine via a two-step protocol [31,32]. Suzuki cross-coupling between boronic ester 3 and bromide 1 yielded L1 in moderate yields [33]. In addition, analysis of the results indicated the formation of a minor amount of the unprotected L1 byproduct. Final coordination was achieved by treating L1 with triphenylborane in dichloromethane at ambient temperature, which generated the diphenylboron complex P1, as confirmed by NMR and mass spectra (Figures S1–S12) [27].

### 2.2. Photophysical Properties

**Figure 1:**
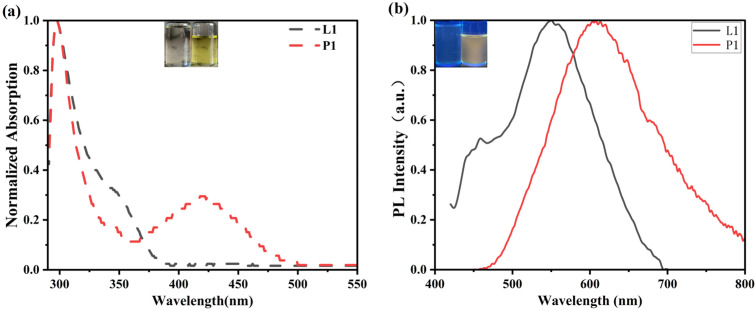
UV-vis spectra and photoluminescence of (**a**) ligands L1 (**b**) and coordinated product P1.

The ultraviolet–visible (UV-vis) absorption and photoluminescence spectra were acquired in toluene solutions at ambient temperature using standardized instrumentation, as detailed in Figure 1. Both the ligand L1 and its boron-coordinated derivative P1 exhibited intense UV-vis absorption bands centered at 298 nm, characteristic of π-π* electronic transitions. Crucially, L1 displayed negligible absorption beyond 380 nm, whereas P1 manifested a distinct charge-transfer absorption band spanning 360–460 nm (Figure 1a), attributed to intramolecular electron transfer between the donor (acridine) and acceptor (coordinated quinoline) moieties. This coordination-induced spectral shift correlated with visible color changes: L1 remained colorless in solution, while P1 exhibited yellow coloration (inset, Figure 1a).

Notably, ligand L1 demonstrated weak fluorescence emission at 553 nm under 365 nm excitation, whereas the coordinated complex P1 emitted intense orange-yellow light at 608 nm (Figure 1b). Spectral quantification revealed a 5-fold enhancement in emission intensity for P1 (80,000 a.u.) relative to L1 (15,000 a.u.). PLQY measurements confirmed this amplification, with PLQY increasing from 0.24% (L1) to 3.4% (P1) (Table 1). These results demonstrated that boron coordination effectively activated luminescence in weakly emissive quinoline derivatives. The 16-fold PLQY enhancement suggested that coordination engineering is a robust means of boosting emission efficiency.

The optical bandgaps derived from the absorption onsets were 3.26 eV for L1 and 2.54 eV for P1 (Table 1). The 0.72 eV P1 bandgap decrease aligned with the observed spectral redshift of P1, confirming coordination-induced electronic modulation.

### 2.3. Theoretical Calculation

**Figure 2:**
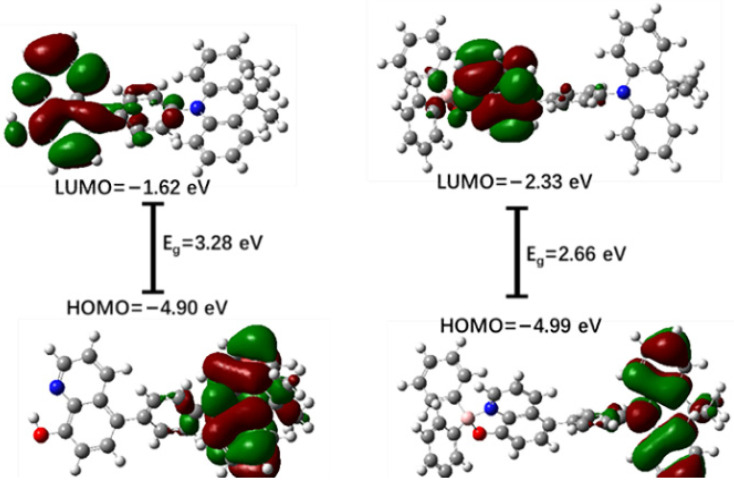
HOMO and LUMO orbital distributions of L1 and P1.

The molecular architecture and frontier orbital characteristics of newly designed compounds L1 and P1 were comprehensively analyzed through computational methods based on DFT. Full structural optimizations were performed using the B3LYP functional with the 6-311G** basis set under gas phase conditions. As depicted in Figure 2, the HOMOs predominantly resided on the electron-donating dimethylacridine moieties in both systems, while the LUMOs of L1 exhibited dual localization on the electron-accepting hydroxyquinoline unit and the central phenyl group. Coordination-induced electronic reorganization in P1 resulted in the LUMO exclusively confined to the hydroxyquinoline acceptor.

The calculated HOMO energies for L1 and P1 were −4.90 eV and −4.99 eV, respectively, with corresponding LUMO values of −1.62 eV and −2.33 eV. This pronounced LUMO stabilization (0.71 eV shift) in the coordinated species P1 directly correlated with the strong electron-withdrawing nature of the BPh_2_ group, and aligned with established coordination chemistry principles [21,26,29]. The higher LUMO energy and wider bandgap (ΔE_g_ = 3.28 eV) in the precursor L1 imposed a significant activation energy on the photoexcited state transitions, supporting its experimentally observed fluorescence inactivity. The coordination complex P1 simultaneously exhibited a LUMO energy depression and bandgap contraction (ΔE_g_ = 2.66 eV), thus creating favorable conditions for radiative decay that accounted for its distinct fluorescent emission. These computational insights systematically connect electronic structure modifications with macroscopic photophysical behavior of coordination-engineered materials.

### 2.4. Electrochemistry and Annihilation ECL

**Figure 3:**
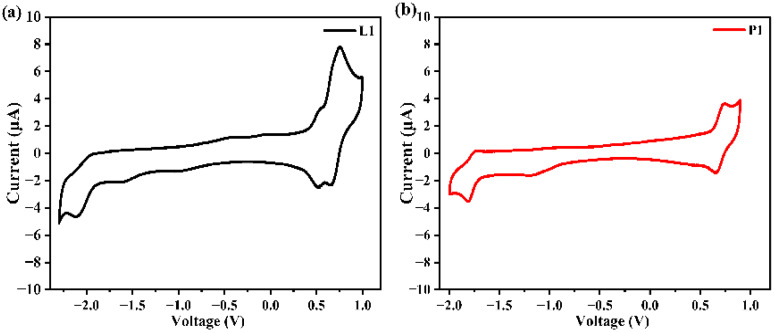
Cyclic voltammograms of (**a**) ligand L1 and (**b**) its boron-coordinated complex P1 in a degassed 1:1 acetonitrile/benzene solution containing 0.1 M TBAP as the supporting electrolyte and Fc/Fc+ as the internal reference.

**Figure 4:**
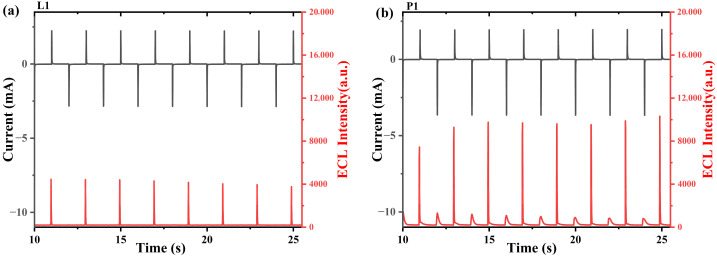
ECL transient curves of (**a**) ligand L1 (0.25 mM) and (**b**) its boron-coordinated complex (0.25 mM) in a 1:1 acetonitrile/benzene solution containing 0.1 M TBAPF_6_.

To elucidate the annihilation ECL characteristics of ligand L1 and its boron coordinated complex P1, cyclic voltammetry was used to establish their redox potentials in a 1:1 acetonitrile/benzene solution containing 0.1 M TBAP, as shown in Figure 3. Scanning toward negative potentials revealed an irreversible reduction of L1 at −2.1 V, while P1 exhibited a quasi-reversible reduction at −1.8 V. This transition from irreversible to quasi-reversible signified enhanced stability of the reduced species upon BPh_2_ coordination, which is critical for efficient ECL. The observed anodic shift in the reduction potential can be attributed to the exceptional electron-accepting ability of boron. During anodic scanning, L1 displayed two quasi-reversible oxidations (0.51 V and 0.75 V), contrasting with the single oxidation of P1 at 0.71 V, indicating that electronic saturation impeded removal of the secondary electron in the coordinated complex. It can be noted that no ECL-voltage curve corresponding to the annihilation process was detected, indicating that their electrochemiluminescence efficiency falls below 0.2% [11]. This is likely attributable to their exceptionally low PLQY. Although intrinsic annihilation ECL was undetectable due to weak signals, synchronized pulsed-potential experiments targeting their primary redox couples successfully captured amplified ECL responses, as demonstrated in Figure 4. These results provided a direct comparison of the charge recombination efficiencies of L1 and P1.

Figure 4 reveals that ligand L1 achieved a maximum ECL intensity of 4000 a.u. when pulsed at its first oxidation and reduction potentials. In stark contrast, the boron-coordinated complex P1 exhibited a significantly amplified ECL response of 10,000 a.u. (2.5-fold enhancement) under identical pulse conditions, which was attributed to boron chelation. This demonstrated that incorporating tetracoordinate boron augmented photoluminescence and substantially enhanced the electrochemiluminescence efficiency. Notably, a distinct ECL behavior emerged between precursor and coordinated species: L1 exhibited exclusive anodic ECL, whereas P1 demonstrated amplified emission at the anode along with newly detected cathodic ECL signals. This dual-electrode activity indicated enhanced stability of the cationic radical species generated upon oxidation in P1.

Cyclic voltammetry revealed critical redox transformations—the irreversible cathodic reduction of L1 evolved into a quasi-reversible process in P1. This type of electrochemical modulation implied that the anionic radical intermediate that had formed during reduction had improved resilience. Consequently, the global ECL enhancement in P1 stemmed from concerted stabilization of both redox-derived radical species through boron coordination.

A significant contribution to the ECL enhancement originates from the coordination-driven rigidification of the molecular framework, which restricts intramolecular motion and thereby diminishes non-radiative energy dissipation. This mechanism aligns with the established rationale often employed to account for ECL amplification. Consequently, coordination-induced ECL enhancement serves as a strategic extension to existing methodologies, such as crystallization- and aggregation-induced ECL enhancement [10,15,34], that collectively leverage the suppression of molecular motion to boost ECL performance.

## 3. Materials and Methods

### 3.1. Reagents and Apparatus

5-Bromo-8-hydroxyquinoline (97%), bis(triphenylphosphine) palladium (II) chloride (PdCl_2_(PPh_3_)_2_, 98%), and triphenylborane (96%) were all obtained from Saen Chemical Technology Co., Ltd. (Shanghai, China). 1-Bromo-4-iodobenzene (98%), sodium tert-butoxide (t-BuONa, 98%), bis(pinacolato)diboron (99%), and bis[(diphenylphosphino)-ferrocence]dichloropalladium (Pd (dppf)Cl_2_, 98%) were purchased from Heowns. The reagents potassium carbonate (99%), cuprous iodide (CuI, 99%), and boron tribromide (99.9%) were bought from Sinopharm Chemical Reagent Co., Ltd. (Shanghai, China). Benzyl bromide (99%), 1-bromo-4-iodobenzene (99%+), tetra-n-butylammonium hexafluoro-phosphate (TBAPF_6_, 99%+), and sodium sulfate anhydrous (Na_2_SO_4_, 99%) were bought from Shanghai Titan Technology Co., Ltd. (Shanghai, China). All of the solvents were purchased from commercial suppliers and used without further purification.

All of the synthetic and analytical procedures were conducted under inert nitrogen conditions unless explicitly stated otherwise, following standard synthetic protocols. Nuclear magnetic resonance (NMR) characterization (^1^H NMR: 400 MHz; ^13^C NMR: 100 MHz) was performed on a Bruker Ascend^TM^ 400 MHz NMR spectrometer (Bruker, Karlsruhe, Germany). Chemical shifts for ^1^H and ^13^C NMR spectra were referenced against tetramethylsilane using residual proton signals from undeuterated solvents. All of the photophysical measurements were obtained using toluene solutions (1 × 10^−4^ M). Absorption spectra were recorded on a UV-1500 spectrophotometer (Macy Instruments, Shanghai, China), and fluorescence emission spectra were captured with a PerkinElmer FL8500 instrument (PerkinElmer, Inc., Shelton, CT, USA). Absolute photoluminescence quantum efficiencies under ambient conditions were quantified using the instrument’s internal sphere accessory. Electrochemical profiling was performed using an HYZ-3002 workstation (HeYongzhong Electronic Technology, Xi’an, China) with a photomultiplier tube at 700 V bias.

### 3.2. Synthesis Process

8-(Benzyloxy)-5-bromoquinoline (**1**)

5-Bromoquinolin-8-ol (0.5 g, 4.46 mmol), benzyl bromide (0.916 g, 5.29 mmol) and potassium carbonate (3.1 g, 22.2 mmol) were added to a dry round-bottom flask. The flask was evacuated and backfilled with Ar three times, and then 10 mL of acetonitrile was added. The mixture was stirred at 85 °C for 12 h and the reaction was monitored by thin-layer chromatography until the starting material was consumed. The mixture was extracted by dichloromethane (DCM) and dried with Na_2_SO_4_. A yellow product (260 mg, 19% yield) was obtained and purified by column chromatography on silica gel using petroleum ether (PE)/DCM at a 1:1 (*v*/*v*) ratio. (Note: according to the literature [28,30], employing benzyl chloride as the reactant can lead to a higher reaction yield.)

10-(4-Bromophenyl)-9,9-dimethyl-9,10-dihydroacridine (**2**)

9,9-Dimethyl-9,10-dihydroacridine (5.0 g, 24.0 mmol), 1-bromo-4-iodobenzene (6.8 g, 24.0 mmol), CuI (46 mg, 0.24 mmol), t-BuONa (3.45 g, 36.0 mmol), and trans-1,2-cyclohexanediamine (0.29 mL, 2.4 mmol) were added to a two-neck round-bottom flask in 1,4-dioxane (100 mL). The mixture was stirred at 100 °C for 24 h, then cooled to room temperature. The crude product was extracted with DCM three times and then purified by column chromatography on silica gel using PE/DCM (3:1), which gave 1 as a white solid (2.5 g, 29% yield).

9,9-Dimethyl-10-(4-(4,4,5,5-tetramethyl-1,3,2-dioxaborolan-2-yl)phenyl)-9,10dihydroacridine (**3**)

Compound **2** (1 g, 2.75 mmol), potassium acetate (2.7 g, 27.5 mmol), bis-(pinacolato)-diboron (1.4 g, 5.5 mmol) and Pd(dppf)Cl_2_ (100.6 mg, 0.05 mmol) were dissolved in a 250-mL two-neck flask with 50 mL of dry dioxane and evacuated for 15 min under Ar. The mixture was heated to 110 °C for 24 h. After reacting, the product was extracted with DCM. Finally, 0.825 g of a white solid (73% yield) was obtained, which was purified by column chromatography (PE/DCM = 5:1, *v*/*v*).

5-(4-(9,9-Dimethylacridin-10(9H)-yl)phenyl)quinolin-8-ol (**L1**)

Compound 3 (157 mg, 0.38 mmol), PdCl_2_(PPh_3_)_2_ (45 mg, 20 mol%), 1 (100 mg, 0.32 mmol) and potassium carbonate (220 mg, 1.6 mmol) were placed in a dry round-bottomed flask, and degassed by flowing Ar for 15 min. At the same time, another flask with 5 mL of DMF and 1 mL of water was degassed by a nitrogen stream for 15 min, and the reactants in the other flask were added. The reaction mixture was transferred to an oil bath and heated to 120 °C for 5 h. After the reaction, the solvent was removed under vacuum distillation. Recrystallization in PE/DCM was used to obtain 50 mg of a yellow solid (L1, 36% yield).

7-(4-(9,9-Dimethylacridin-10(9H)-yl)phenyl)-2,2-diphenyl-2H-2l4,3l4-[1,3,2]oxazaborolo[5,4,3-ij]quinoline (**P1**)

To a solution of L1 (150 mg, 0.35 mmol) in 10 mL of dichloromethane, a 0.25 mM BPh_3_ solution (4.2 mL, 1.05 mmol) was added. The mixture was stirred at room temperature to react for 24 h. The solvent was removed and the crude product was purified by column chromatography on silica gel using PE: DCM at a 1:1 ratio (*v*/*v*) to obtain P1 (77 mg, 37%).

### 3.3. Calculations

Electronic structures and molecular conformations were modeled computationally. Ground-state optimizations employed density functional theory (DFT) via Gaussian 16 software, utilizing the B3LYP functional with 6-31G(d) basis set. This approach optimizes precision/efficiency trade-offs for organic frameworks. Calculations initiated from pre-optimized geometries used default convergence thresholds.

### 3.4. Electrochemistry and ECL Measurement

For ECL measurements, solutions containing 0.25 mM analyte and 0.1 M TBAP in mixture of acetonitrile/benzene (1:1) were fabricated under inert atmosphere. Teflon-sealed cells ensured oxygen-free conditions during electrochemical characterization. The three-electrode configuration featured a 3-mm glassy carbon working electrode alongside platinum counter and reference electrodes. Ferrocene provided potential calibration standards. All glassware and electrodes used in the measurements were rigorously cleaned following established protocols, the details of which can be found in our previously published work [11,12]. Note: Due to its toxic nature, benzene requires strict adherence to safety practices, including the use of appropriate engineering controls (e.g., fume hoods) and personal protective equipment.

## 4. Conclusions

In summary, a molecule comprising acridine and 8-hydroxyquinoline yielded a weakly emissive scaffold (L1), whose coordination to a BPh_2_ group dramatically stabilized the LUMO level and induced a pronounced emission redshift in complex P1. Spectroscopic analyses revealed a 16-fold enhancement in photoluminescence quantum yield for P1, attributed to the suppression of non-radiative decay via coordination-driven rigidification of the molecular framework. Electrochemical studies demonstrated that the optimized boron geometry also stabilizes radical intermediates during redox cycling, leading to a 2.5-fold enhancement in ECL intensity. DFT calculations confirmed that HOMO-LUMO energy gaps are effectively tuned by boron coordination. This BPh_2_-coordination strategy presents a generalizable molecular design approach for modulating the optoelectronic properties of donor-acceptor systems, with promising potential for developing advanced ECL sensors and OLED emitters. Nevertheless, we acknowledge that the absolute PLQY of P1 (3.4%) remains modest compared to state-of-the-art emitters, indicating the need for further molecular optimization to achieve practical application viability. Future work will focus on extending this design principle to other molecular architectures with improved emission efficiencies and exploring their integration into functional devices.

## Figures and Tables

**Table 1:** Summary of photophysical and electrochemical properties of L1 and P1.

Compounds	λ_abs_ [nm]	λ_FL_ [nm]	Φ_PL_^1^ [%]	Φ_ECL_^2^ [%]	ΔE_g_^3^ [eV]
L1	298	553	0.24%	/	3.26
P1	298, 420	608	3.4%	<0.2%	2.54

^1^ Absolute PLQYs evaluated under air (and argon). ^2^ 0.2% from a compound whose annihilation ECL efficiency could be barely measured [11]. ^3^ Optical band gaps were estimated from the onset wavelengths of the UV–vis using the formula E_g_ = 1240/λ_onset, absorption_.

## Data Availability

The original contributions presented in this study are included in the article/supplementary material. Further inquiries can be directed to the corresponding authors.

## Supplementary Materials

The following supporting information can be downloaded at: https://www.mdpi.com/article/10.3390/ijms262110528/s1.

## References

[1] Hesari M., Ding Z. (2016). Review—Electrogenerated Chemiluminescence: Light Years Ahead. J. Electrochem. Soc..

[2] Dong J., Feng J. (2023). Electrochemiluminescence from Single Molecule to Imaging. Anal. Chem..

[3] Zhang H., Jiang H., Liu X., Wang X. (2024). A review of innovative electrochemical strategies for bioactive molecule detection and cell imaging: Current advances and challenges. Anal. Chim. Acta.

[4] Shen K.-Y., Zhan J., Shen L., Xiong Z., Zhu H.-T., Wang A.-J., Yuan P.-X., Feng J.-J. (2023). Hydrogen Bond Organic Frameworks as Radical Reactors for Enhancement in ECL Efficiency and Their Ultrasensitive Biosensing. Anal. Chem..

[5] Bruno J.G., Kiel J.L. (2002). Use of magnetic beads in selection and detection of biotoxin aptamers by electrochemiluminescence and enzymatic methods. Biotechniques.

[6] Kijek T.M., Rossi C.A., Moss D., Parker R.W., Henchal E.A. (2000). Rapid and sensitive immunomagnetic-electrochemiluminescent detection of staphyloccocal enterotoxin B. J. Immunol. Methods.

[7] Shelton Daniel R., Karns Jeffrey S. (2001). Quantitative Detection of Escherichia coli O157 in Surface Waters by Using Immunomagnetic Electrochemiluminescence. Appl. Environ. Microbiol..

[8] Kerr E., Doeven E.H., Barbante G.J., Hogan C.F., Hayne D.J., Donnelly P.S., Francis P.S. (2016). New perspectives on the annihilation electrogenerated chemiluminescence of mixed metal complexes in solution. Chem. Sci..

[9] Fernandez-Hernandez J.M., Longhi E., Cysewski R., Polo F., Josel H.-P., De Cola L. (2016). Photophysics and Electrochemiluminescence of Bright Cyclometalated Ir(III) Complexes in Aqueous Solutions. Anal. Chem..

[10] Wong J.M., Zhang R., Xie P., Yang L., Zhang M., Zhou R., Wang R., Shen Y., Yang B., Wang H.-B. (2020). Revealing Crystallization-Induced Blue-Shift Emission of a Di-Boron Complex by Enhanced Photoluminescence and Electrochemiluminescence. Angew. Chem. Int. Ed..

[11] Cheng J., Yang L., Wang R., Wisner J.A., Ding Z., Wang H.-B. (2024). Intensified electrochemiluminescence and photoluminescence via supramolecular anion recognition interactions. Chem. Sci..

[12] Zhou X., Cheng J., Wang H. (2025). Synthesis and Electrochemiluminescence of a Di-Boron Thermally Activated Delayed Fluorescence Emitter. Molecules.

[13] Hesari M., Ding Z. (2017). A Grand Avenue to Au Nanocluster Electrochemiluminescence. Anal. Chim. Acta.

[14] Qin Y., Wang Z., Xu J., Han F., Zhao X., Han D., Liu Y., Kang Z., Niu L. (2020). Carbon Nitride Quantum Dots Enhancing the Anodic Electrochemiluminescence of Ruthenium(II) Tris(2,2′-bipyridyl) via Inhibiting the Oxygen Evolution Reaction. Anal. Chem..

[15] Zhu Z., Zeng C., Zhao Y., Ma J., Yao X., Huo S., Feng Y., Wang M., Lu X. (2023). Precise Modulation of Intramolecular Aggregation-induced Electrochemiluminescence by Tetraphenylethylene-based Supramolecular Architectures. Angew. Chem. Int. Ed..

[16] Tang C.W., VanSlyke S.A. (1987). Organic electroluminescent diodes. Appl. Phys. Lett..

[17] Li X., Zhang G., Song Q. (2023). Recent advances in the construction of tetracoordinate boron compounds. Chem. Commun..

[18] Mellerup S.K., Wang S. (2019). Boron-based stimuli responsive materials. Chem. Soc. Rev..

[19] Wakamiya A., Taniguchi T., Yamaguchi S. (2006). Intramolecular B–N Coordination as a Scaffold for Electron-Transporting Materials: Synthesis and Properties of Boryl-Substituted Thienylthiazoles. Angew. Chem. Int. Ed..

[20] Cui L.X., Horioka A., Ishimatsu R., Mamada M., Adachi C., Tahara K., Hoshino Y., Ono T. (2024). Advanced Molecular Design for Efficient Multicolor Electrochemiluminescence and Amplified Spontaneous Emission Based on Tetra-BF2 Complexes. Adv. Opt. Mater..

[21] Hu D., Huang R., Fang Y. (2025). Recent Advances in Tetra-Coordinate Boron-Based Photoactive Molecules for Luminescent Sensing, Imaging, and Anticounterfeiting. Precis. Chem..

[22] Jin C., Yang X., Zhao W., Zhao Y., Wang Z., Tan J. (2024). Synthesis, properties and emerging applications of multi-boron coordinated chromophores. Coord. Chem. Rev..

[23] Anthony J.E. (2008). The Larger Acenes: Versatile Organic Semiconductors. Angew. Chem. Int. Ed..

[24] Liu Z., Meng L., Jiang Y., Li C., Gu H., Zhao K., Zhang J., Meng H., Ren Y. (2025). Hyperconjugation Engineering of π-Extended Azaphosphinines for Designing Tunable Thermally Activated Delayed Fluorescence Emitters. J. Am. Chem. Soc..

[25] Fukazawa A., Yamada H., Yamaguchi S. (2008). Phosphonium- and borate-bridged zwitterionic ladder stilbene and its extended analogues. Angew. Chem. (Int. Ed. Engl.).

[26] Li D., Zhang H., Wang Y. (2013). Four-coordinate organoboron compounds for organic light-emitting diodes (OLEDs). Chem. Soc. Rev..

[27] Hellstrom S.L., Ugolotti J., Britovsek G.J.P., Jones T.S., White A.J.P. (2008). The effect of fluorination on the luminescent behaviour of 8-hydroxyquinoline boron compounds. New J. Chem..

[28] Li Z., Yu L., Yuan J., Ye Y., He Y., Huang B., Gao X. (2017). 8-Hydroxy Quinoline Derivatives as Auxiliary Ligands for Red-Emitting Cyclic-Platinum Phosphorescent Complexes: Synthesis and Properties. Helv. Chim. Acta.

[29] Rao Y.L., Wang S. (2011). Four-coordinate organoboron compounds with a π-conjugated chelate ligand for optoelectronic applications. Inorg. Chem..

[30] Pohl R., Montes V.A., Shinar J., Anzenbacher P. (2004). Red−Green−Blue Emission from Tris(5-aryl-8-quinolinolate)Al(III) Complexes. J. Org. Chem..

[31] Park H.-J., Han S.H., Lee J.Y. (2017). Molecular Design of Thermally Activated Delayed-Fluorescent Emitters Using 2,2′-Bipyrimidine as the Acceptor in Donor–Acceptor Structures. Chem.—Asian J..

[32] Ji H., Wu L.-Y., Cai J.-H., Li G.-R., Gan N.-N., Wang Z.-H. (2018). Room-temperature borylation and one-pot two-step borylation/Suzuki–Miyaura cross-coupling reaction of aryl chlorides. RSC Adv..

[33] Li G., Zhan F., Lou W., Wang D., Deng C., Cao L., Yang Y., Zhang Q., She Y. (2020). Efficient deep-blue organic light-emitting diodes employing difluoroboron-enabled thermally activated delayed fluorescence emitters. J. Mater. Chem. C.

[34] Carrara S., Aliprandi A., Hogan C.F., De Cola L. (2017). Aggregation-Induced Electrochemiluminescence of Platinum(II) Complexes. J. Am. Chem. Soc..

