# Supplementary material for: Synthesis and Properties of a Novel Four-Coordinate 8-Hdroxy-Quinolate-Based Complex
Source: Int J Mol Sci. 2025 Oct 29;26(21):10528. doi: 10.3390/ijms262110528 (PMC12607314; doi:10.3390/ijms262110528)
Supplement: Supplementary file 1 [file ijms-26-10528-s001.zip › ijms-3922431-supplementary.pdf]

8-(Benzyloxy)-5-bromoquinoline (1)

^{1}H NMR (400 MHz, $CDCl_3$) δ 9.00 (dd, J = 4.2, 1.5 Hz, 1H), 8.50 (dd, J = 8.6, 1.6 Hz, 1H), 7.63 (d, J = 8.4 Hz, 1H), 7.55 (dd, J = 8.6, 4.2 Hz, 1H), 7.50 (d, J = 7.2 Hz, 2H), 7.37 (t, J = 7.3 Hz, 2H), 7.30 (t, J = 7.3 Hz, 1H), 6.91 (d, J = 8.4 Hz, 1H), 5.44 (s, 2H). ^{13}C NMR (101 MHz, $CDCl_3$) δ 154.19 (s), 149.85 (s), 141.25 (s), 136.45 (s), 135.57 (s), 129.97 (s), 128.72 (s), 128.40 (s), 128.03 (s), 127.14 (s), 122.70 (s), 112.22 (s), 110.54 (s), 70.96 (s).

10-(4-Bromophenyl)-9,9-dimethyl-9,10-dihydroacridine (2)

^{1}H NMR (400 MHz, $CDCl_3$) δ 7.82–7.70 (m, 2H), 7.46 (dd, J = 7.5, 1.7 Hz, 2H), 7.25–7.19 (m, 2H), 6.96 (dqd, J = 14.5, 7.3, 1.4 Hz, 4H), 6.25 (dd, J = 8.0, 1.2 Hz, 2H), 1.68 (s, 6H). ^{13}C NMR (101 MHz, $CDCl_3$) δ 140.61 (s), 140.34 (s), 134.22 (s), 133.23 (s), 130.12 (s), 126.48 (s), 125.33 (s), 122.10 (s), 120.82 (s), 113.90 (s), 35.98 (s), 31.24 (s).

9,9-Dimethyl-10-(4-(4,4,5,5-tetramethyl-1,3,2-dioxaborolan-2-yl)phenyl)-9,10dihydroacridine (3)

^{1}H NMR (400 MHz, $CDCl_3$) δ 8.10–8.02 (m, 2H), 7.44 (dd, J = 7.3, 2.2 Hz, 2H), 7.37–7.31 (m, 2H), 6.91 (qd, J = 7.2, 1.9 Hz, 4H), 6.25 (dd, J = 7.5, 1.8 Hz, 2H), 1.69 (s, 6H), 1.40 (s, 12H). ^{13}C NMR (101 MHz, $CDCl_3$) δ 144.03 (s), 140.74 (s), 137.33 (s), 130.65 (s), 129.97 (s), 126.34 (s), 125.22 (s), 120.54 (s), 114.11 (s), 84.13 (s), 35.99 (s), 31.34 (s), 24.96 (s).

5-(4-(9,9-Dimethylacridin-10(9H)-yl)phenyl)quinolin-8-ol (L1)

^{1}H NMR (400 MHz, CDCl3) δ 8.87 (m, 1H), 8.45 (d, J = 8.5 Hz, 1H), 7.72 (d, J = 7.4 Hz, 2H), 7.60 (d, J = 7.4 Hz, 1H), 7.56–7.37 (m, 6H), 7.32 (d, J = 7.7 Hz, 1H), 7.02 (dt, J = 30.8, 7.2 Hz, 4H), 6.44 (d, J = 8.0 Hz, 2H), 1.74 (s, 6H). ^{13}C NMR (101 MHz, $CDCl_3$) δ 151.81 (s), 147.65 (s), 140.77 (s), 140.11 (s), 139.16 (s), 138.19 (s), 134.33 (s), 132.28 (s), 131.23 (s), 129.95 (s), 129.72 (s), 128.46 (s), 126.30 (d, J = 20.0 Hz), 125.08 (s), 121.84 (s), 120.48 (s), 113.88 (s), 109.39 (s), 35.83 (s), 31.02 (s). HRMS: calcd for $C_{30}H_{25}N_2O$ $[M + H]^+$, 429.1967; found $[M + H]^+$, 429.1977.

7-(4-(9,9-Dimethylacridin-10(9H)-yl)phenyl)-2,2-diphenyl-2H-2l4,3l4-[1,3,2]oxazaborolo[5,4,3-ij] quinoline (P1)

^{1}H NMR (400 MHz, $CDCl_3$) δ 8.68 (dd, J = 13.1, 6.7 Hz, 2H), 7.81 (t, J = 6.6 Hz, 1H), 7.76–7.66 (m, 3H), 7.59–7.43 (m, 9H), 7.35–7.27 (m, 6H), 7.06–7.00 (m, 2H), 6.96 (t, J = 7.3 Hz, 2H), 6.39 (t, J = 7.5 Hz, 2H), 1.72 (s, 6H). ^{13}C NMR (101 MHz, $CDCl_3$) δ 158.58 (s), 140.91 (s), 140.65 (s), 139.57 (s),

137.95 (d, J = 22.2 Hz), 137.69 (s), 133.54 (s), 132.14–131.74 (m), 130.21 (s), 127.67 (s), 127.10 (s), 126.71 (s), 126.39 (s), 125.29 (d, J = 6.4 Hz), 123.05 (s), 120.75 (s), 114.04 (s), 109.77 (s), 36.04 (s), 31.20 (s). HRMS: calcd for $C_{42}H_{34}BN_2O$ $[M + H]^+$, 593.2764; found $[M + H]^+$, 593.2768.

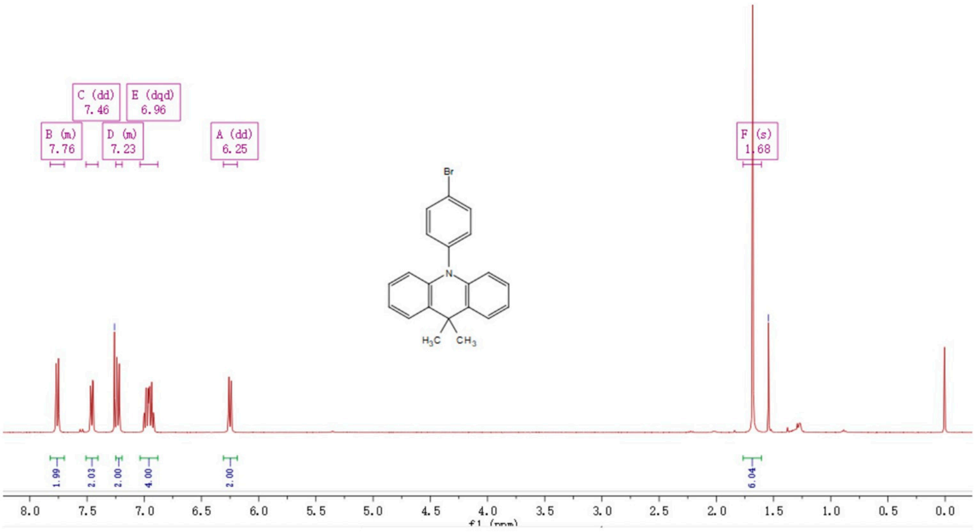

Figure S1. ^{1}H NMR spectrum of intermediate 1.

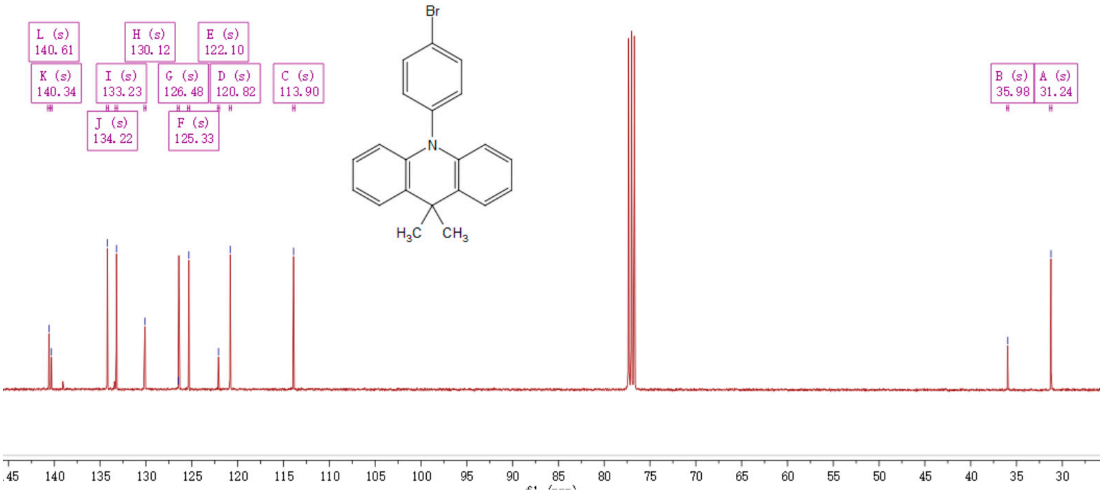

Figure S2. ^{13}C NMR spectrum of intermediate 1.

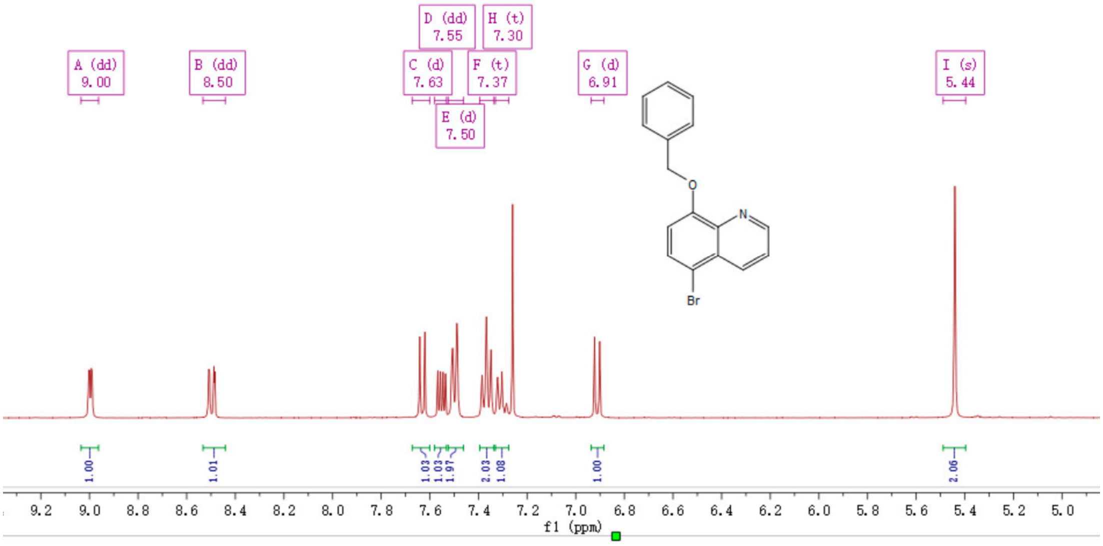

Figure S3. ^{1}H NMR spectrum of intermediate 2.

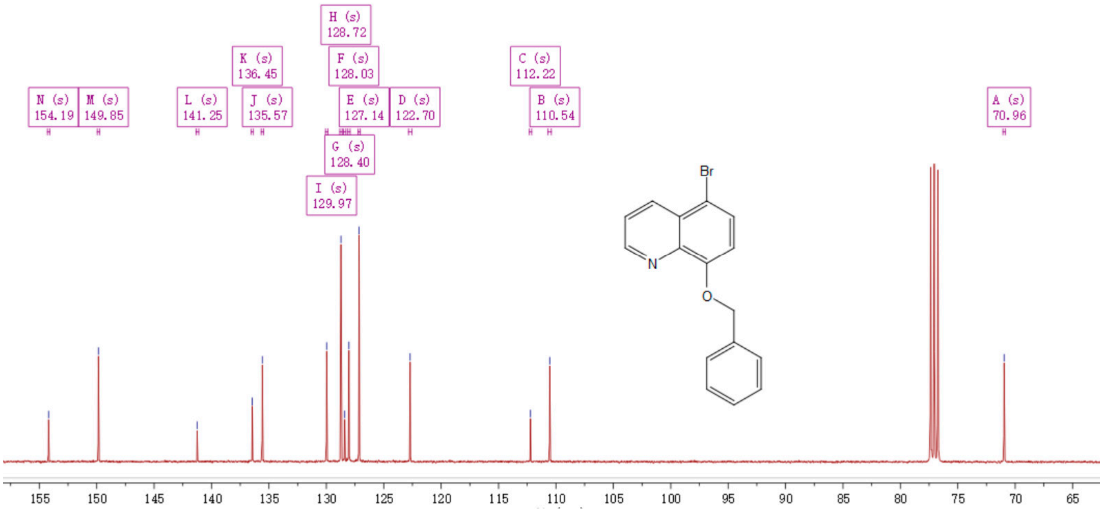

Figure S4. ^{13}C NMR spectrum of intermediate 2.

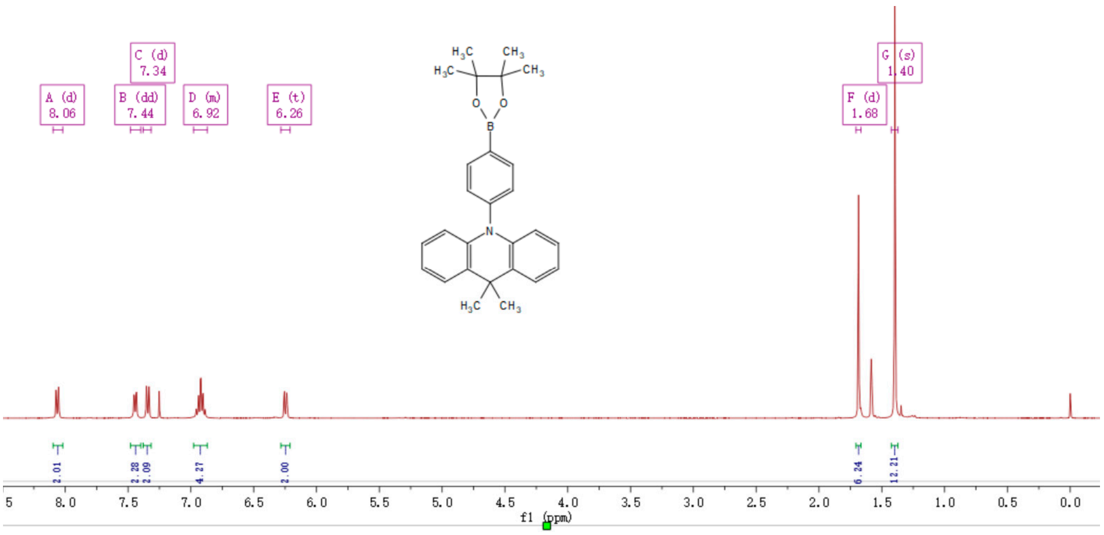

Figure S5. ^{1}H NMR spectrum of intermediate 3.

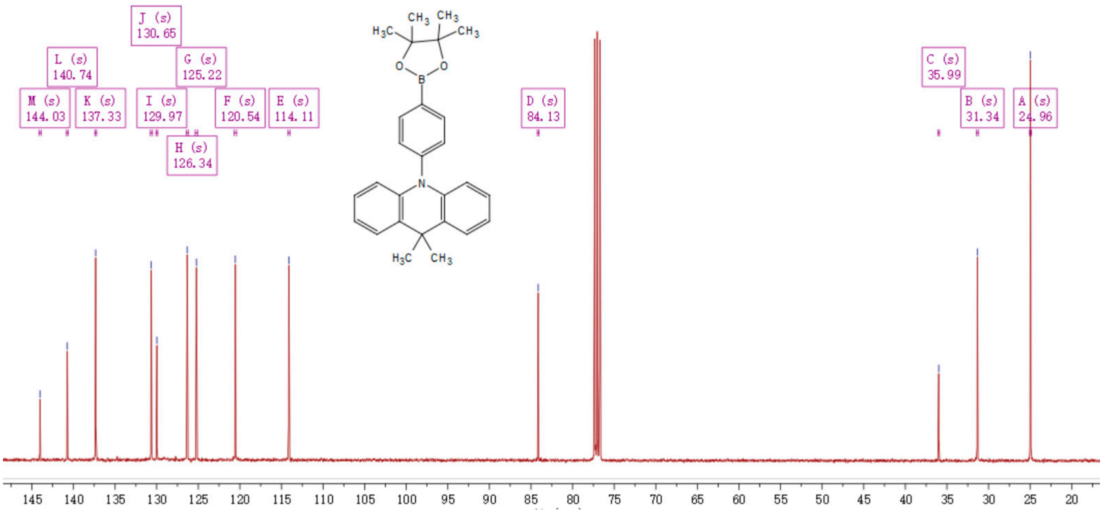

Figure S6. ^{13}C NMR spectrum of intermediate 3.

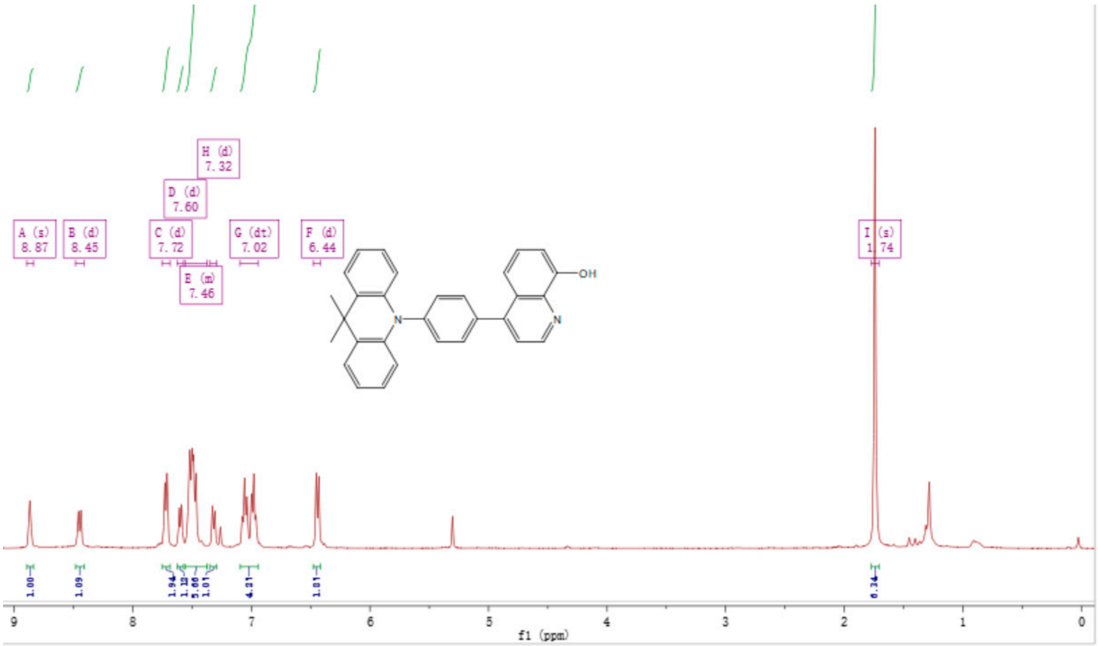

Figure S7. ^{1}H NMR spectrum of L1.

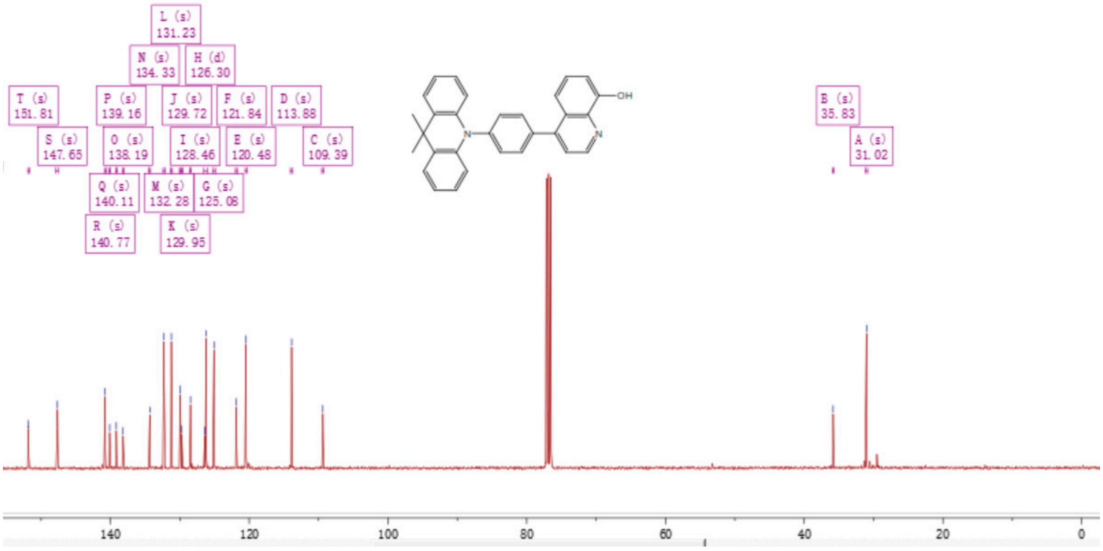

Figure S8. ^{13}C NMR spectrum of L1.

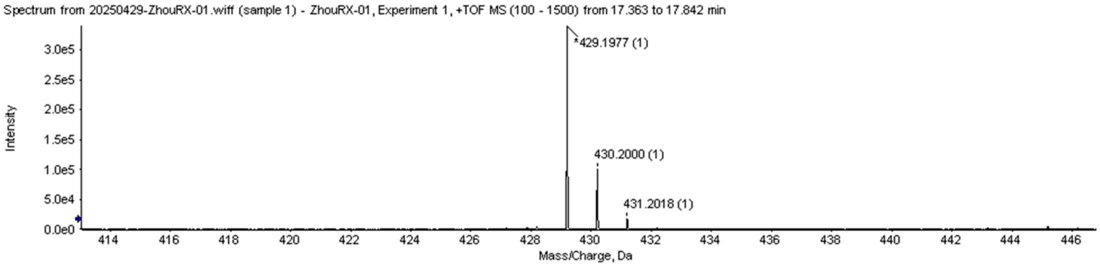

Figure S9. Mass spectrum of L1.

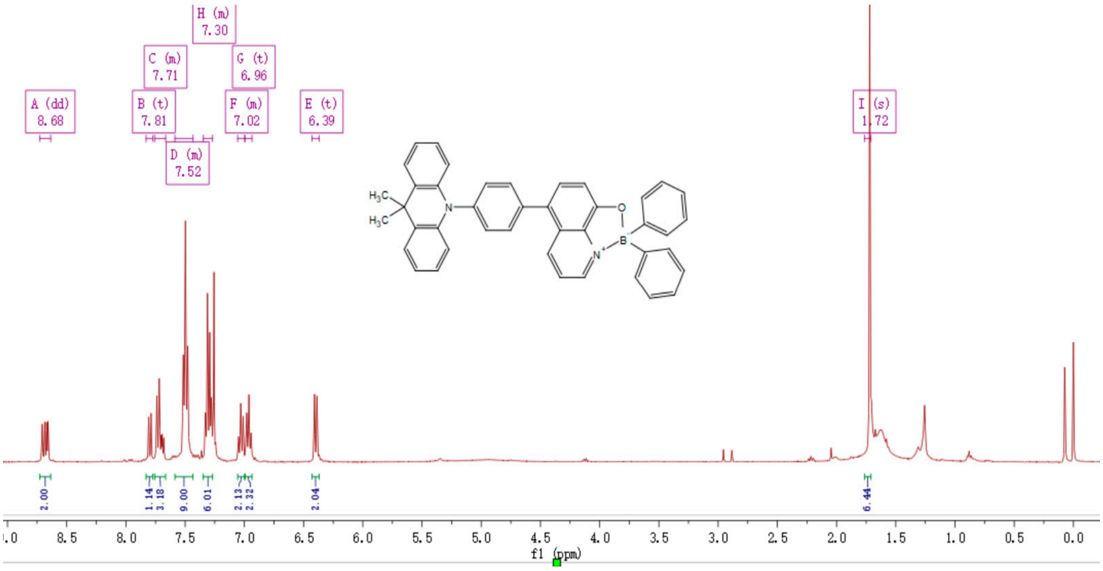

Figure S10. ^{1}H NMR spectrum of P1.

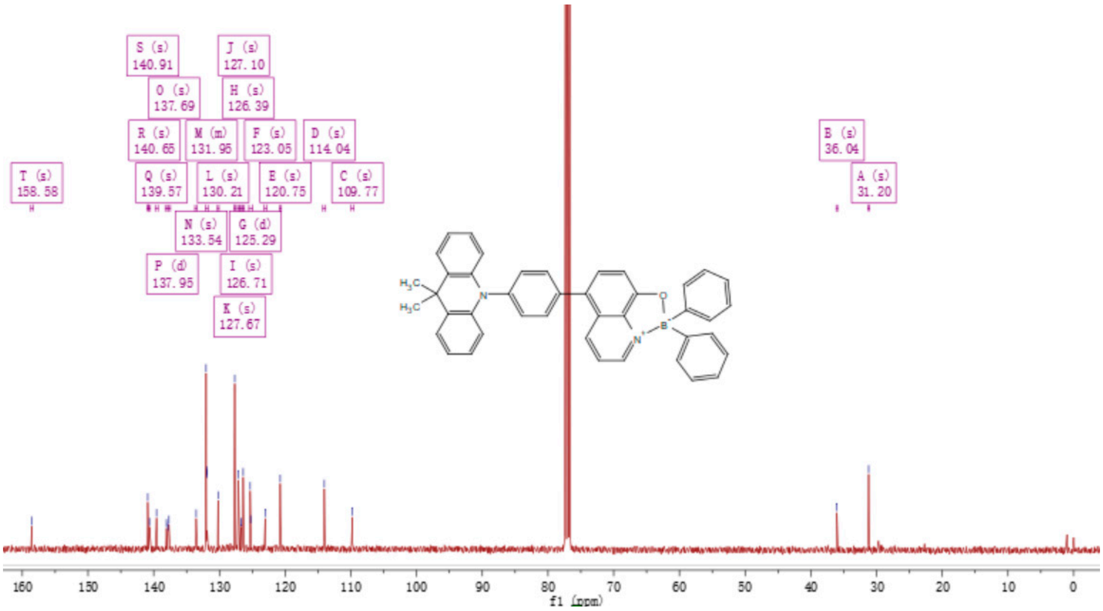

Figure S11. ^{13}C NMR spectrum of P1.

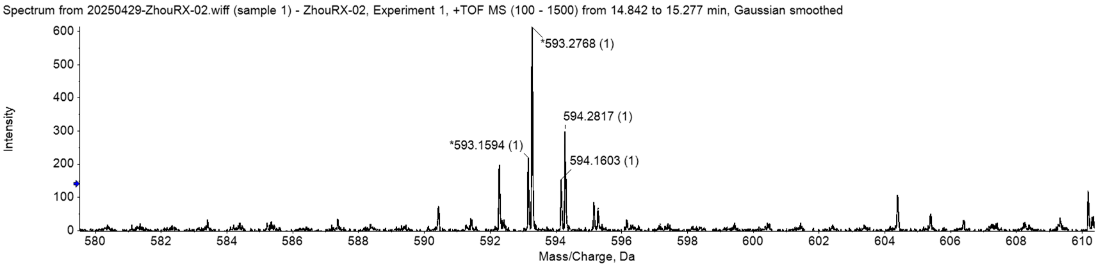

Figure S12. Mass spectrum of P1.

Table S1. The PLQY and onset wavelengths of the UV-vis absorption of **L1**.

**Times**	**PLQY /%**	$\lambda_{\text{onset, UV-vis absorption}}$
**1**	0.16	388
**2**	0.25	381
**3**	0.31	374
**Average**	0.24	380

Table S2. The PLQY and onset wavelengths of the UV-vis absorption of **P1**.

**Times**	**PLQY /%**	$\lambda_{\text{onset, UV-vis absorption}}$
**1**	3.1	496
**2**	3.3	485
**3**	3.8	484
**Average**	3.4	488
